# Bioinformatics gene analysis for potential biomarkers and therapeutic targets of Parkinson’s disease based on neutrophil extracellular traps

**DOI:** 10.3389/fnagi.2024.1388226

**Published:** 2024-05-31

**Authors:** Qiang Wang, Youquan Gu, Jun Chen, Xiaoyan Liu, Chen Xie, Xueping Wang

**Affiliations:** ^1^Department of Neurology, The First Hospital of Lanzhou University, Lanzhou, China; ^2^Chengdu shi Longquanyi qu Diyi Renmin Yiyuan: The First People’s Hospital of Longquanyi District, Longquanyi District, Chengdu, China

**Keywords:** neutrophil extracellular traps, Parkinson’s disease, GPR 78, CADM3, CACNA1E, bioinformatics

## Abstract

**Introduction:**

Neutrophil extracellular traps (NETs) provide key innate immune mechanisms, and studies have shown innate immunity and adaptive immunity are directly linked to Parkinson’s disease (PD) pathology. However, limited research has been conducted on NETs in the context of PD.

**Methods:**

A differential analysis was implemented to acquire differentially expressed genes (DEGs) between PD and control as well as between high- and low-score groups determined by a gene set variation analysis (GSVA). Then, the genes within the critical module, obtained through a weighted gene co-expression network analysis (WGCNA), were intersected with the DEGs to identify the overlapping genes. Then, five kinds of algorithms in the protein–protein interaction (PPI) were performed to identify potential biomarkers. Subsequently, a nomogram for forecasting PD probability was created. An enrichment analysis and an immune infiltration analysis were performed on the identified biomarkers. qRT-PCR was performed to validate the expression trends of three biomarkers.

**Results:**

We revealed 798 DEGs between PD and control groups as well as 168 DEGs between high- and low-score groups obtained by differential analyses. The pink module containing 926 genes was identified as the critical module. According to the intersection of these gene sets, a total of 43 overlapping genes were screened out. Furthermore, GPR78, CADM3, and CACNA1E were confirmed as biomarkers. Moreover, we found that biomarkers mainly participated in pathways, such as the ‘hydrogen peroxide catabolic process’, and ‘cell cycle’; five kinds of differential immune cells between PD and control groups were identified. Finally, the qRT-PCR analysis demonstrated the up-regulation of GPR78, CADM3, and CACNA1E in the PD group.

**Discussion:**

Our study authenticated GPR78, CADM3, and CACNA1E as the biomarkers associated with PD. These findings provide an original reference for the diagnosis and treatment of PD.

## 1 Introduction

Parkinson’s disease (PD) is a progressive neurodegenerative disorder characterized by the deterioration of motor activities. This deterioration results from the impairment of the dopaminergic nigrostriatal system causing the primary motor symptoms, including static tremor, bradykinesia, rigidity, and postural instability. These symptoms can arise from both genetic and environmental risk factors (Kalia and Lang, 2015). The characteristic pathological change of PD was aggregation of intraneuronal α-synuclein known as Lewy bodies (LB). Research by Andrei Surguchov and Alexei Surguchev found that synucleins, small intrinsically disordered proteins prone to aggregation, are implicated in both neurodegenerative diseases and cancer (Surguchov and Surguchev, 2022). The onset of the disease predated the first clinical symptom for many years. However, the precise etiology of dopaminergic cell death remains elusive. While 5–10% of PD cases have a genetic basis, resulting from mutations in genes such as SNCA encoding alpha-synuclein, DJ-1, PINK, and LRRK2, leading to early onset of PD, the majority of cases are idiopathic and associated with aging. In addition to genetic predisposition, other risk factors included environmental toxins, pesticides, heavy metals, traumatic lesions, and bacterial or viral infections (Wirdefeldt et al., 2011), all of which are closely associated with inflammation. Research studies have demonstrated that neuroinflammation contributed significantly to the pathophysiology of PD and was intricately linked to both its onset and progression (Araújo et al., 2022).

Previous studies have identified shared genetic variants among PD patients and other autoimmune and inflammatory disorders, including Crohn’s disease, further supporting the involvement of the immune system in PD pathogenesis (Witoelar et al., 2017; Hui et al., 2018). Moreover, exposure to environmental insecticides has been shown to enhance immune responses in individuals carrying HLA-DR variants, thereby increasing the risk of developing PD by 2.48-fold (Kannarkat et al., 2015). However, the precise trigger for inflammation in PD remains unclear. In addition to the extensively documented microgliosis and astrogliosis in PD brains, peripheral inflammation and PD risk-associated genes substantiate a significant contribution of the chronic inflammatory response to the progression of this neurodegenerative disorder.

Neutrophil extracellular traps (NETs) are intricate structures composed of chromatin filaments coated with histones, proteases, and granular and cytosolic proteins. The process known as NETosis refers to the production and release of these NETs by neutrophils. NETosis facilitates the immobilization and capture of bacteria, fungi, or viruses by neutrophils, thereby enhancing the efficient elimination of pathogens. Although the formation of NETs is a key bactericidal mechanism, emerging research has demonstrated that NETs can also elicit detrimental effects on the human body. Recently, emerging evidence suggests that NETs may play a significant role in the pathogenesis of various non-infectious diseases, such as systemic lupus erythematosus (SLE), rheumatoid arthritis (RA), diabetes, atherosclerosis, vasculitis, thrombosis, cancer, wound healing, and trauma. The release of NETs can damage the host tissue, promote the development of autoimmunity, and give rise to other dysfunctional outcomes, including metastasis, thrombosis, and aberrant coagulation. The NET formation has been reported to play a role in the pathophysiological processes of various brain injuries (Vaibhav et al., 2020). Zenaro et al. (2015) recently demonstrated the generation of endovascular NETs in an animal model of Alzheimer’s disease (AD), resulting in the disruption of the blood–brain barrier (BBB). Additionally, intravascular NET-induced thrombosis may exacerbate cerebral amyloid angiopathy, a distinctive feature of AD resulting from Aβ deposits.

Despite there has been an extensive understanding of the pathogenesis and epidemiology of PD, the etiology remains elusive, and no definitive cure or preventive therapy has yet been discovered (Kalia and Lang, 2015; Blauwendraat et al., 2020). The diagnosis of PD, however, remains a challenge due to the overlapping clinical features with other neurodegenerative conditions and the lack of definitive diagnostic tests or biomarkers in the early stages. The findings collectively indicate that the immune system and inflammation play a crucial role in the development of PD (Calabrese et al., 2018; Mészáros et al., 2020). Net-induced inflammatory changes are implicated in a range of neurodegenerative alterations. Hence, this study aimed to identify NET-associated gene biomarkers through a bioinformatics gene analysis as a foundation for early diagnosis of Parkinson’s disease.

## 2 Materials and methods

### 2.1 Data sources

**Figure 1:**
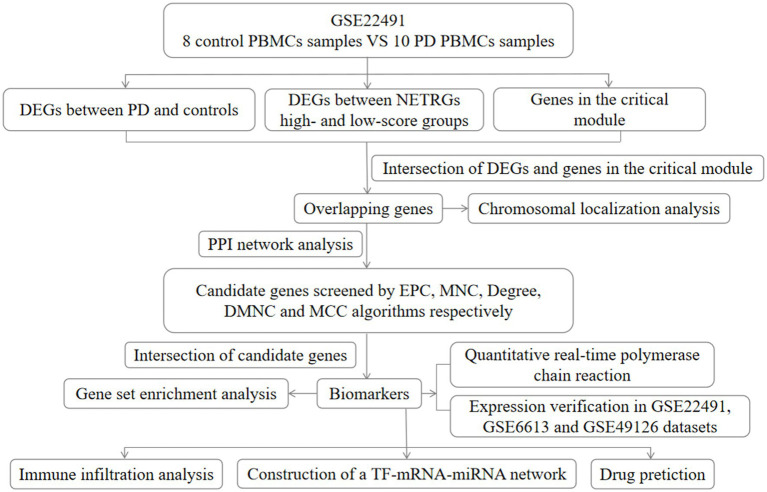
Flow chart of this study. DEG, differentially expressed gene; PD, Parkinson’s disease; EPC, percolated component; MNC, maximum neighborhood component; DMNC, density of maximum neighborhood component; MCC, maximal clique centrality; NETRGs, neutrophil extracellular trap-related genes.

The datasets of PD were achieved through the GEO database. The GSE22491 dataset (GPL6480, Whole Human Genome Microarray 4x44K G4112F) comprised microarray data from 8 control samples and 10 PD samples, and it was utilized for the training set. Moreover, there were RNA-sequencing (RNA-seq) data from 22 control samples and 50 PD samples in the GSE6613 dataset (GPL96 [HG-U133A], Affymetrix Human Genome U133A Array). The GSE49126 dataset (GPL4133, Agilent-014850 Whole Human Genome Microarray 4x44K G4112F) contained microarray data from 20 control samples and 30 PD samples. The GSE6613 and GSE49126 datasets were utilized as external validation sets. The samples in the GSE22491 and GSE49126 datasets were peripheral blood mononuclear cells (PBMC) samples, and the samples in the GSE6613 dataset were whole blood samples. In addition, 136 NET-related genes (NETRGs) were extracted based on the references after removing the repetitions (Wu et al., 2022). The flowchart of this study is listed in Figure 1.

### 2.2 Differential expression analysis and enrichment analysis

In the GSE22491 dataset, DEGs between PD and control groups were acquired by the limma (v 3.52.4) (Ritchie et al., 2015) package (*p*.value <0.05, |log_2_FC| > 1). We adopted Agilent software to process the data before the differential expression analysis. Then, the heat maps and volcano maps of differentially expressed genes (DEGs) between PD and control groups were plotted by pheatmap (v 1.0.12) and ggplot2 (v 3.3.6) (Ito and Murphy, 2013) packages, respectively. For further observing and investigating the items and signaling pathways that these DEGs were involved in, then the Gene ontology (GO) (p.adjust <0.05) and Kyoto Encyclopedia of Genes and Genomes (p.value <0.05) enrichment analyses were performed using clusterProfiler (v 4.4.4) package (Wu et al., 2021). According to the expression levels of NETRGs, the score of each sample was computed using the gene set variation analysis (GSVA). (v 1.46.0) package (Hänzelmann et al., 2013), and then, the samples were classified into high- and low-score groups based on the median GSVA score. Subsequently, the hallmark pathway scores of two score samples were computed using the GSVA (v 1.46.0) package (Hänzelmann et al., 2013). The differences in pathway scores between the two score groups were compared by the Wilcoxon test method. Meanwhile, the DEGs between the two score groups were acquired by the limma (v 3.52.4) (Ritchie et al., 2015) package (p.value <0.05, |log2FC| > 1).

### 2.3 The weighted gene co-expression network analysis (WGCNA) and screening of overlapping genes

The WGCNA was performed on all samples in the training set to screen the critical module. First, outlier samples were eliminated to secure the precision of the analysis by sample clustering. An appropriate soft threshold (β) was selected to make sure that the engagement between genes conformed to the scale-free distribution to the maximum extent. Then, different modules were obtained by the dynamic tree-cutting algorithm. Subsequently, the sample grouping (control and PD) was utilized as traits, and the correlation analysis was utilized to evaluate the relationships between modules and the traits. Finally, the module that most related to the traits was defined as the critical module. Furthermore, the DEGs between PD and control groups, DEGs between two score groups, and the genes in the critical module were overlapped to achieve the overlapping genes. In addition, the chromosomal localization analysis of overlapping genes was conducted using the RCircos package (Zhang et al., 2013).

### 2.4 Identification and verification of biomarkers

Based on the above overlapping genes, the protein–protein interaction (PPI) network was created. Then, percolated component (EPC), maximum neighborhood component (MNC), degree, density of maximum neighborhood component (DMNC), and maximal clique centrality (MCC) algorithms were performed to acquire the candidate genes, and then, the top 5 genes in the five algorithms were selected as the candidate genes. Furthermore, the top 5 genes in those five algorithms were intersected to screen out biomarkers. In addition, the Wilcoxon test method was conducted to compare the differences in the expression of biomarkers between control and PD groups in the training set, GSE6613, and GSE49126 datasets. In addition, according to the above biomarkers, a nomogram for forecasting the disease probability of PD patients was created. Moreover, a calibration curve was drawn to evaluate the precision of the model.

### 2.5 Enrichment analysis

To investigate the related biological functions and pathways of the biomarkers, moreover, the ‘c5.go.v2022.1.Hs.entrez.gmt’ and ‘c2.cp.kegg.v7.5.1.symbols.gmt’ were utilized as the background gene set, and then, the gene set enrichment analysis (GSEA) was conducted using the clusterProfiler (v 4.4.4) package (Wu et al., 2021) (|NES| > 1, NOM *p* < 0.05, and q < 0.25). Furthermore, in order to further understand the molecular mechanism of biomarkers, the classical signaling pathway analysis was performed by the ingenuity pathway analysis (IPA) to explore the signaling pathways that were significantly affected by the biomarkers (*p* < 0.05). Moreover, the relationships between biomarkers and other diseases or functions were analyzed.

### 2.6 Immune infiltration analysis

In order to further explore the immune infiltration condition, in the GSE22491 dataset, the CIBERSORT algorithm was implemented to analyze the infiltration abundance of the immune cells in every PBMC sample. Furthermore, the differential immune cells between PD and control groups were identified by the *t*-test (*p* < 0.05). Moreover, the relationships between biomarkers and differential immune cells were computed by the Spearman method.

### 2.7 The regulation network and the drug prediction

The miRNAs corresponding to the above biomarkers were forecasted using the miRDB,^1^ miRmap,^2^ and miRWalk^3^ online databases. The miRNAs in those three databases were crossed to acquire the common miRNAs (co-miRNAs) of each biomarker. Moreover, the TFs of the biomarkers were forecasted by the ChIP-X Enrichment Analysis 3 (ChEA3)^4^ online database. The TF–mRNA–miRNA regulatory network was created. Furthermore, based on the co-miRNAs of each biomarker, the lncRNAs were forecasted using the StarBase online tool.^5^ In addition, the potential drugs of those above biomarkers were acquired through the drug–gene interaction database DGIdb (www.dgidb.org) and CTD^6^ database. Moreover, the mRNA–drug network was created.

### 2.8 Power analysis

To evaluate the adequacy of the sample size, we conducted a power analysis utilizing the R package pwr (Version 1.3–0) to estimate the sample size based on biomarkers. The significance level was set at 0.05, with a desired statistical power level of 0.9. The effect size for the *t*-test (Cohen’s *d*) was computed using RNA-seq data.

### 2.9 Quantitative real-time PCR verification

The blood samples were obtained from patients with knowledge and consent from The First Hospital of Lanzhou University, and this study was approved by The First Hospital of Lanzhou University ethics committee. There were five PD samples and five control samples. Total RNA from blood samples was isolated and purified by TRIzol (Ambion) reagent following the instruction manual. Then, the extracted RNA was tested for concentration by NanoPhotometer N50. Then, reverse transcription was performed utilizing SureScript-First-strand-cDNA-synthesis-kit (Servicebio) with an ordinary PCR instrument to synthesize cDNA. Reverse transcription product cDNA was diluted 5–20 times with ddH2O (RNase/DNase free). Subsequently, polymerase chain reaction (PCR) amplification reaction was performed by CFX96 real-time quantitative PCR instrument: 1 min at 95°C (pre-denaturation), followed by at 95°C for 20 s (denaturation), 55°C for 20 s (annealing) and 72°C for 30 s (elongation). The above reactions were subjected to 40 cycles. Primer sequences are shown in Table 1.

**Table 1:** Primer sequences for quantitative real-time PCR verification of three co-DEGs.

Primer	Sequence
GPR78 F	ATGGGACTCTCTGATGGGCT
GPR78 R	ATGCCAAGAGCAAGTGACGA
CADM3 F	AGCAGACTCTCTACTTTGGGG
CADM3 R	GCACAGGCATAGTGAAGATTGA
CACNA1E F	ATTCAACAGTTCACAGCGGC
CACNA1E R	GCGAGCCATCCTGAGGTTTA
internal reference–GAPDH F	CGAAGGTGGAGTCAACGGATTT
internal reference–GAPDH R	ATGGGTGGAATCATATTGGAAC

DEG, Differentially expressed gene.

## 3 Results

### 3.1 Acquisition of DEGs

**Figure 2:**
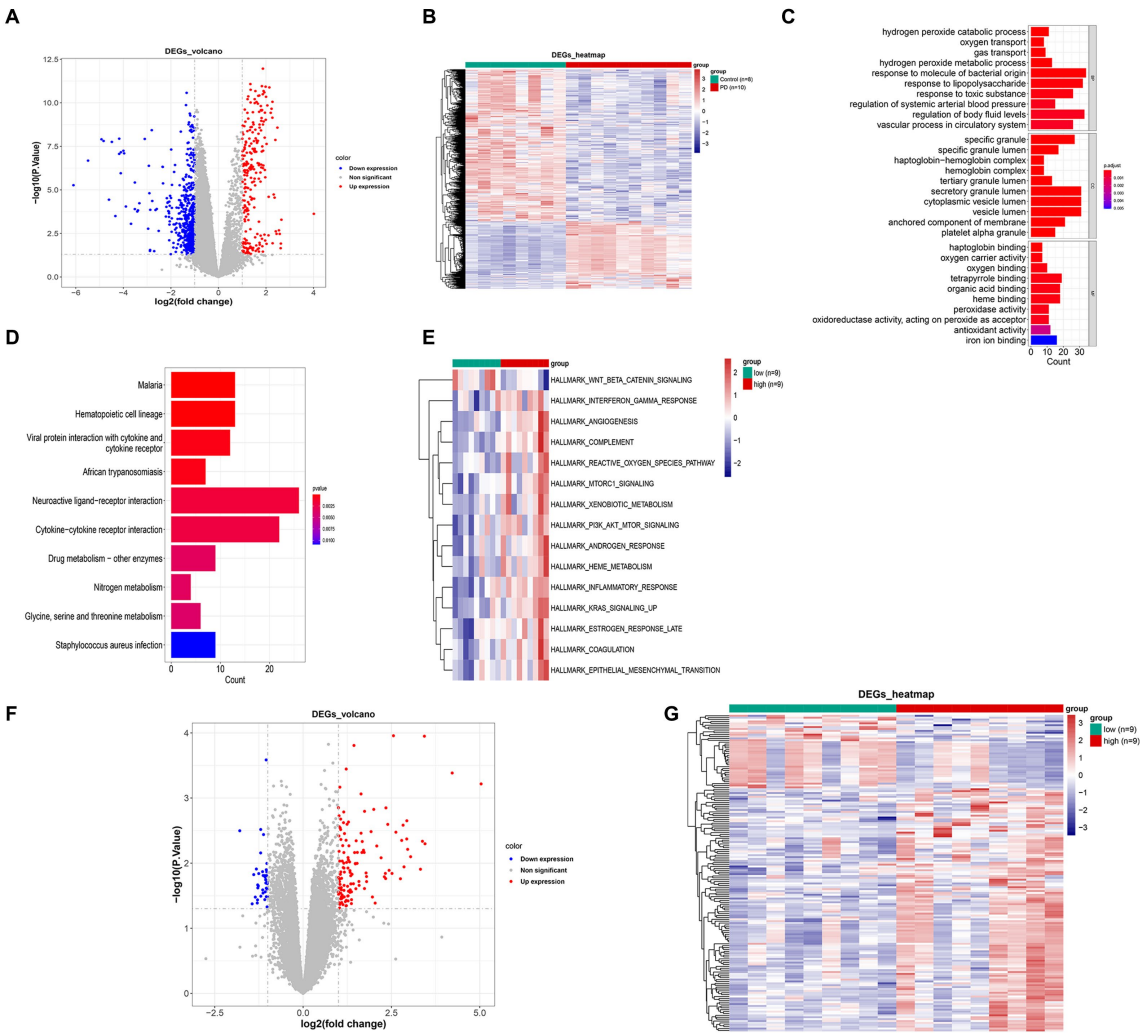
Hierarchical clustering analysis of differentially expressed genes between PD and control groups. **(A)** Volcano map of differentially expressed genes. **(B)** Expression heat map of PD-associated DEGs. **(C)** GO enrichment analysis of DEGs. **(D)** KEGG enrichment analysis of DEGs. **(E)** Differential path heat map between high- and low-GSVA score groups. **(F)** Volcano map of differentially expressed genes between high- and low-GSVA score groups. **(G)** Expression heat map of DEGs between high- and low-GSVA score groups. GO, Gene Ontology; KEGG, Kyoto Encyclopedia of Genes and Genomes; DEG, differentially expressed gene; PD, Parkinson’s disease.

In the GSE22491 dataset, there were 798 DEGs between PD and control groups, including 245 up-regulated DEGs and 553 down-regulated DEGs (Figure 2A; Supplementary Table S1). The expression heat map of PD-associated DEGs is shown in Figure 2B. The functional enrichment analysis revealed that the DEGs between the PD and control groups were predominantly enriched in processes related to ‘hydrogen peroxide catabolic process’, ‘specific granule’, ‘haptoglobin binding’, etc. (Figures 2C,D; Supplementary Tables S2–S3). The high- and low-score groups exhibited significant disparities in 15 pathways, including ‘WNT beta catenin signaling’, ‘angiogenesis’, ‘reactive oxygen species pathway’, and other hallmark pathways. These findings indicate functional differences between the two score groups (Figure 2E). A total of 168 DEGs between high- and low-score groups were identified among which 245 DEGs were up-regulated and 553 DEGs were down-regulated (Figure 2F; Supplementary Table S4). The expression heat map of DEGs in the two score groups is shown in Figure 2G.

### 3.2 Acquisition of critical module and overlapping genes

**Figure 3:**
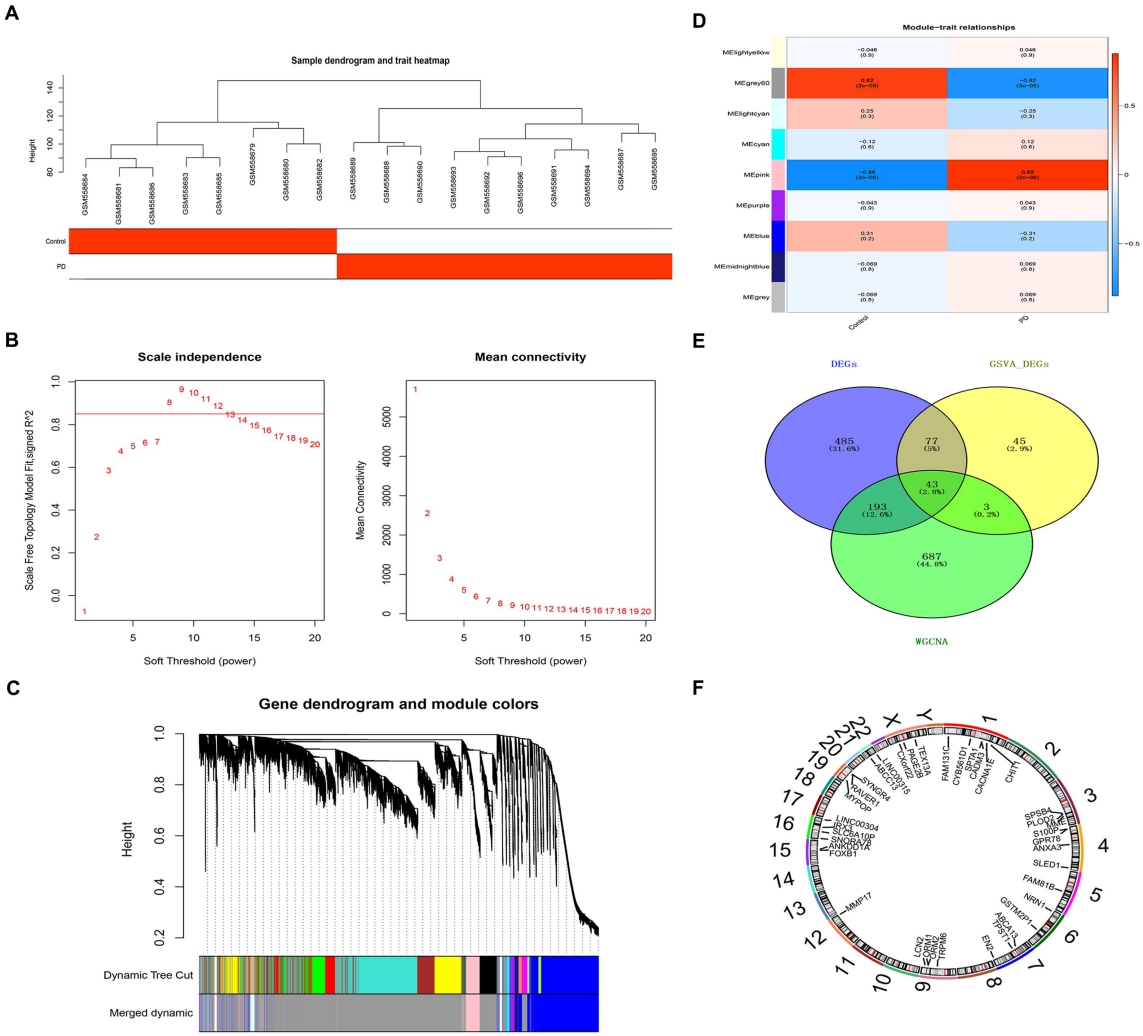
Critical module and overlapping genes between PD and NETs. **(A)** Sample clustering diagram. **(B)** Selection of soft threshold β. **(C)** Module cluster diagram. **(D)** Heat map of the relationship between gene modules and traits within sample groups. **(E)** Venn diagram of candidate genes. **(F)** Mapping of candidate genes on chromosomes. PD, Parkinson’s disease; NET, neutrophil extracellular traps.

The sample clustering result indicated there was no outlier sample (Figure 3A); β was 8, indicating that those genes conform to a scale-free distribution to the greatest extent possible (Figure 3B); nine modules were obtained after merging (Figure 3C). The pink module, containing 926 genes, was identified as the critical module (|Cor| = 0.88 and *p* = 2e-06) (Figure 3D). According to the intersection, 43 overlapping genes were screened out (Figure 3E). The overlapping genes were found on chromosomes 1, 3, 4, 5, 6, 7, 9, 12, 15, 16, 19, 21 and the sex chromosome. For instance, GPR78 was located on chromosome number four (Figure 3F).

### 3.3 Three biomarkers were identified

**Figure 4:**
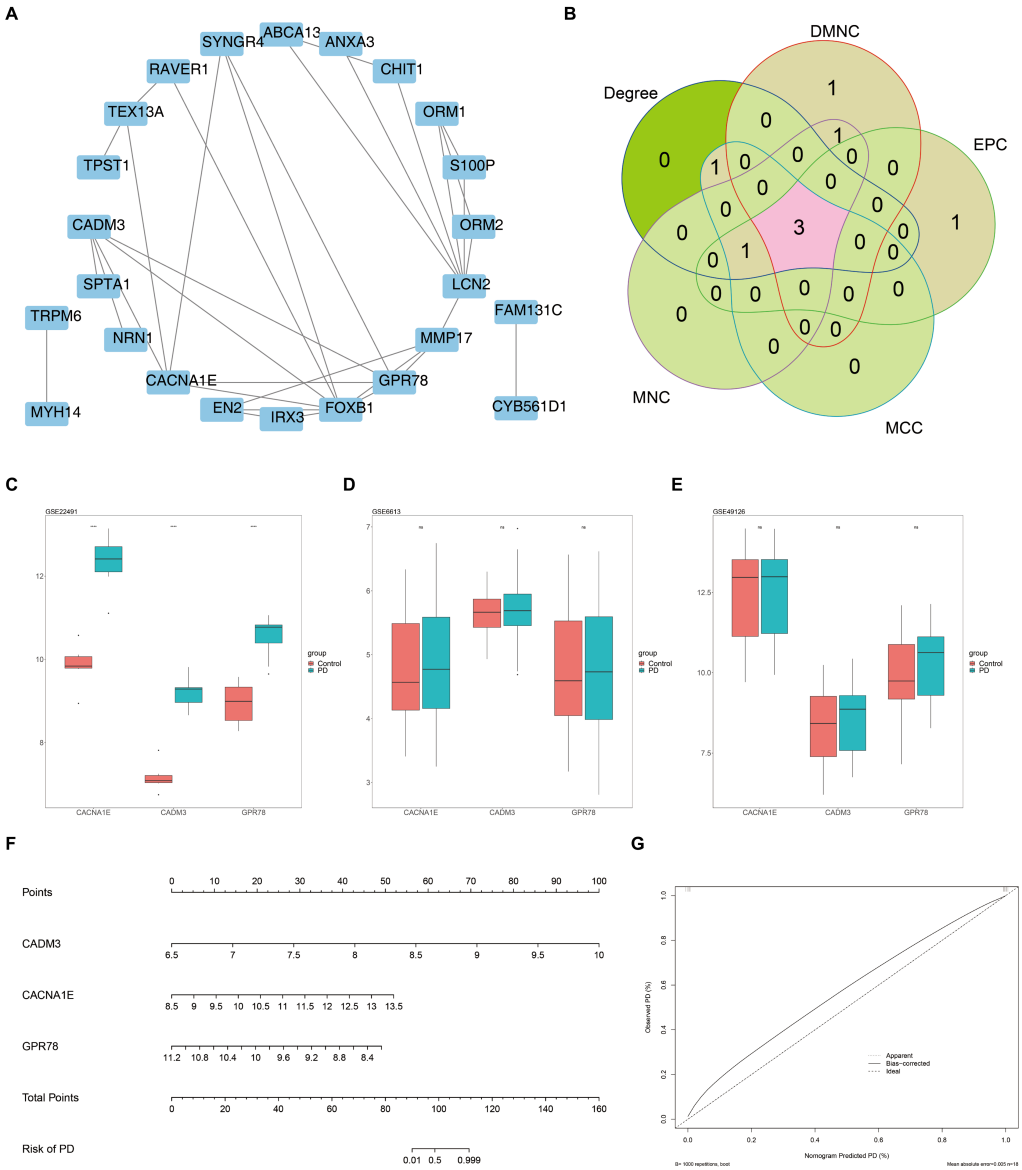
Three key DEGs were identified as biomarkers for PD. **(A)** Construction of candidate gene PPI network. **(B)** Venn diagram of gene intersection is generated by applying five algorithms. A total of three biomarkers including GPR78, CADM3, and CACNA1E were identified. **(C)** Validation of key gene expression in training set GSE22491. **(D)** Validation of key gene expression in training set GSE6613. **(E)** Validation of key gene expression in training set GSE49126. **(F)** Nomogram for disease diagnostic prediction of the PD patients was created based on GPR78, CADM3, and CACNA1E. **(G)** Calibration curve of the nomogram. PD, Parkinson’s disease; DEG, differential expression gene; GPR 78, orphan G protein-coupled receptor 78; CADM3, cell adhesion molecule 3.

There were 24 overlapping genes in the PPI network, and GPR78 had a higher connectivity degree (Figure 4A). A total of three biomarkers including GPR78, CADM3, and CACNA1E were identified (Figure 4B). In addition, in the training set, these three biomarkers were all up-regulated in the PD group, and they were all significantly different between the two groups (Figure 4C). Meanwhile, in the external validation sets GSE6613 and GSE49126, the expression trends of the three biomarkers were the same as those in the training set, which had good universality (Figures 4D,E). A nomogram for disease diagnostic prediction of the PD patients was created based on GPR78, CADM3, and CACNA1E (Figure 4F). The calibration curve was plotted based on the above nomogram, demonstrating that the predictive ability of the nomogram was favorable (Figure 4G).

### 3.4 The GSEA of the biomarkers

**Figure 5:**
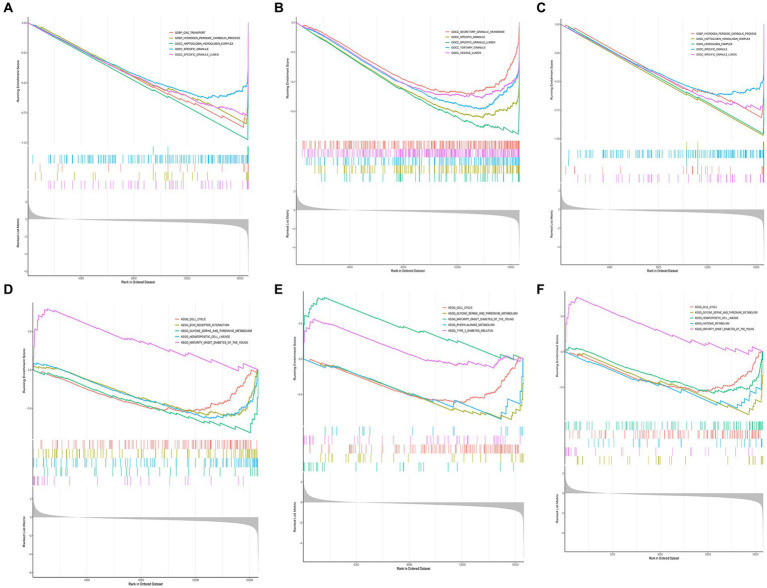
GSEA enrichment analysis of key DEGs. **(A)** GO enrichment analysis of CACNA1E gene. **(B)** GO enrichment analysis of CADM3 gene. **(C)** GO enrichment analysis of GPR78 gene. **(D)** KEGG enrichment analysis of CACNA1E gene. **(E)** KEGG enrichment analysis of CADM3 gene. **(F)** KEGG enrichment analysis of GPR78 gene. DEG, differentially Expressed Gene; GO, Gene Ontology; KEGG, Kyoto Encyclopedia of Genes and Genomes; GPR 78, orphan G protein-coupled receptor 78; CADM3, cell adhesion molecule 3.

We performed GSEA on the aforementioned biomarkers. According to the results of GO enrichment analysis, we observed that GPR78 and CACNA1E primarily participated in biological processes such as ‘hydrogen peroxide catabolic process’ and molecular complexes like ‘haptoglobin hemoglobin complex’. Moreover, the KEGG enrichment analysis demonstrated that GPR78, CADM3, and CACNA1E were mainly associated with ‘cell cycle’ and ‘glycine serine and threonine metabolism’ KEGG pathways. (Figures 5A–F; Supplementary Tables S5–S10).

### 3.5 Ingenuity pathway analysis of biomarkers

**Figure 6:**
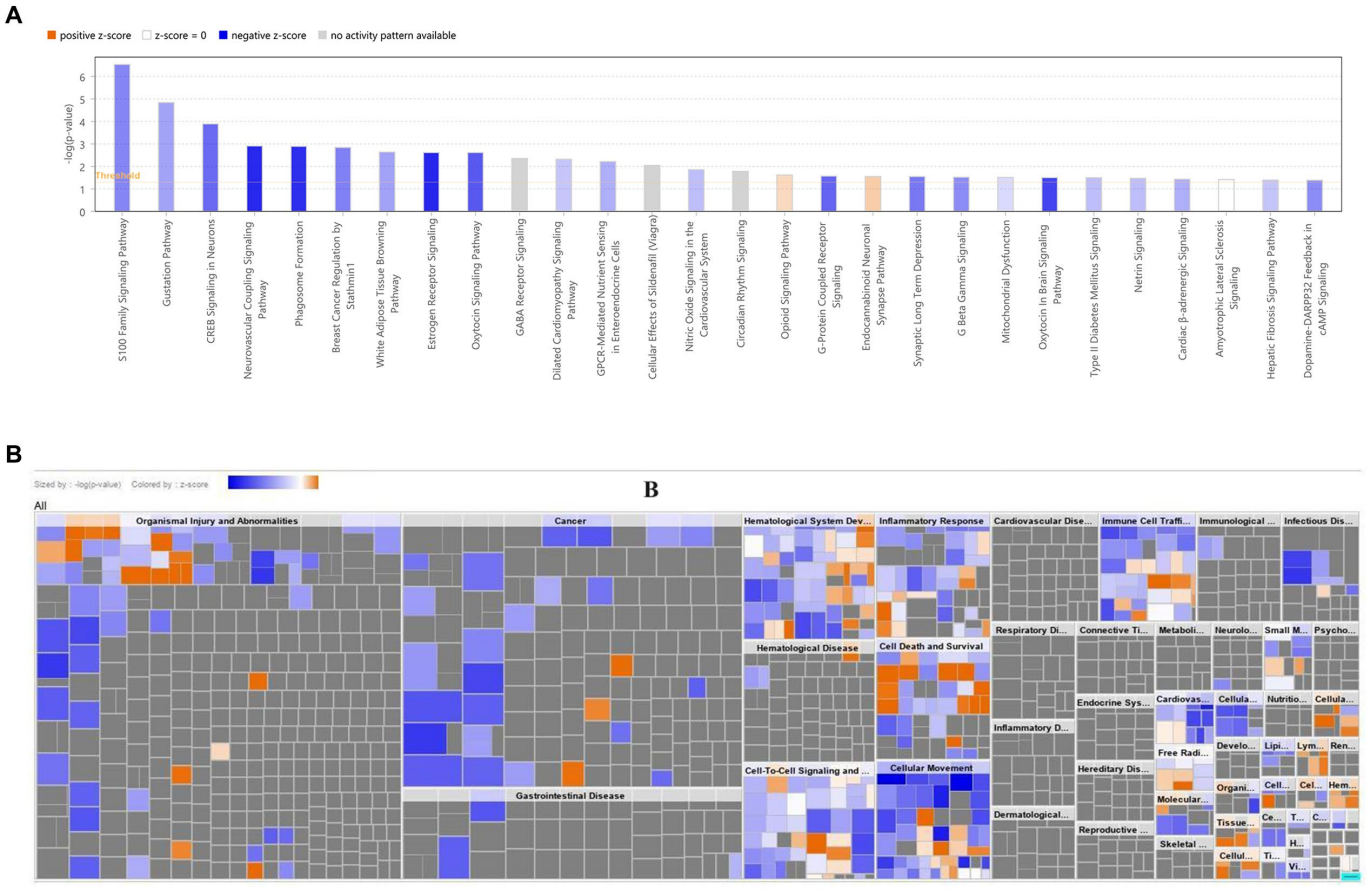
Disease and functional enrichment analysis of key DEGs. **(A)** Classical pathway analysis of key DEGs. **(B)** Heat map of key DEGs on disease biological functions. The orange zone represents the activated pathway, and the darker the color, the greater the absolute value of the z-score; blue zone represents the suppressed pathway, and the darker the color, the greater the absolute value of the z-score. DEG, Differential expressed gene.

The IPA results revealed that biomarkers were primarily associated with the ‘S100 Family Signaling Pathway’, ‘Neurovascular Coupling Signaling Pathway’, and other related pathways (Figure 6A; Supplementary Table S11). The biomarkers were involved in ‘Cell Death and Survival’, ‘Inflammatory Response’, etc. biological functions (Figure 6B).

### 3.6 Immune infiltration analysis between PD and control groups

**Figure 7:**
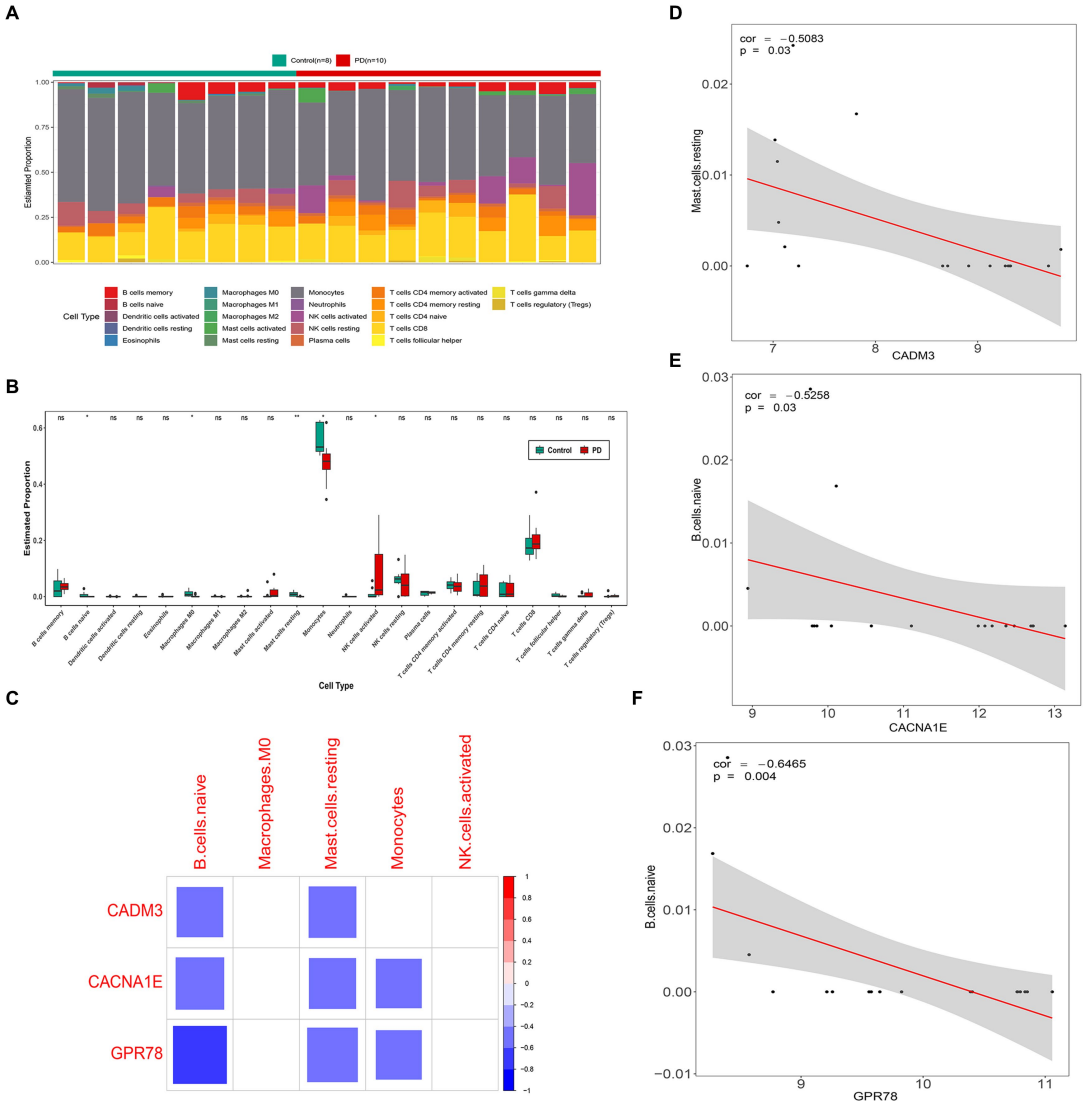
Immune infiltration analysis between PD and control groups. **(A)** Heat map of immune cell infiltration. **(B)** Box plots of 22 types of immune cell infiltration in PD and control groups. **(C)** Correlation analysis of key genes and differential immune cells. The blank squares in the figure indicate that the correlation between genes and immune cells is not significant (*p* ≥ 0.05), and the remained colored squares indicate that the correlation is significant (*p* < 0.05). Red represents a positive correlation, blue represents a negative correlation, and the darker the color, the stronger the correlation. **(D)** Scatter plot of correlation between CACNA1E and naive B cells. **(E)** Scatterplot of correlation between CADM3 and resting mast cells. **(F)** Scatter plot of correlation between GPR78 and naïve B cells. PD, Parkinson’s disease; GPR 78, orphan G protein-coupled receptor 78; CADM3, cell adhesion molecule 3.

The immune cell infiltration level is displayed in Figure 7A. There were five kinds of differential immune cells (resting mast cells, macrophages M0, monocytes, naive B cells, and activated NK cells) between PD and control groups (Figure 7B). We could observe that GPR78, CADM3, and CACNA1E were all negatively correlated with two differential immune cells (naive B cells and resting mast cells) (|Cor| > 0.3) and GPR78 had the highest negatively associated with naive B cells (|Cor| = 0.6465; Figures 7C–F).

### 3.7 The miRNA–mRNA–TF and ceRNA networks

**Figure 8:**
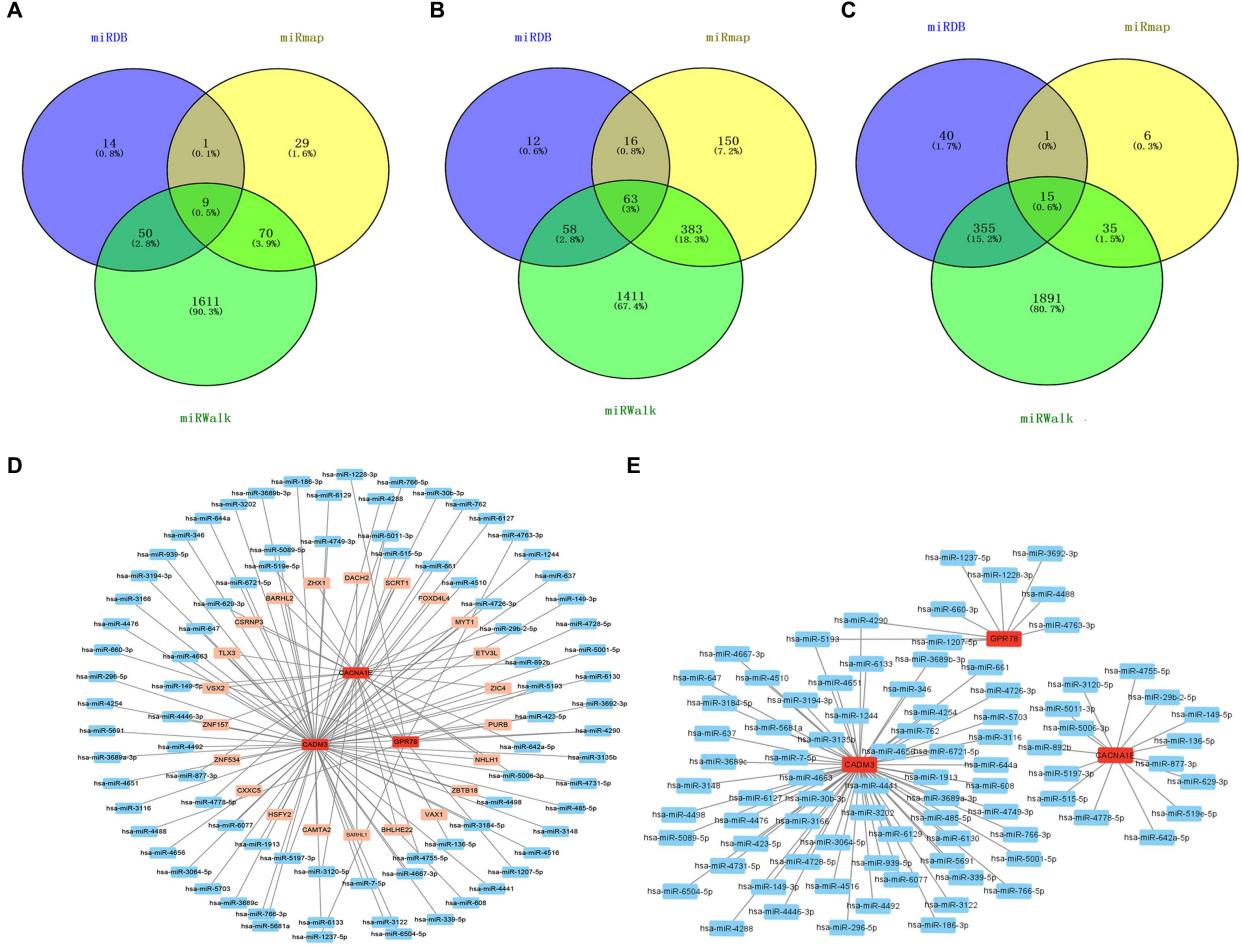
miRNA–mRNA–TF and ceRNA networks. **(A)** Venn diagram of GPR78 gene predicts miRNA intersection. **(B)** Venn diagram of CADM3 gene predicts miRNA intersection. **(C)** Venn diagram of CACNA1E gene predicts miRNA intersection. **(D)** TF–mRNA–miRNA regulatory network. Red is mRNA; orange is TF; blue is miRNA. **(E)** mRNA–miRNA regulatory network. Red is mRNA; blue is miRNA. GPR 78, orphan G protein-coupled receptor 78; CADM3, cell adhesion molecule 3.

According to the intersection, there were 9 miRNAs, 63 miRNAs, and 15 miRNAs forecasted based on GPR78, CADM3, and CACNA1E, respectively, and a total of 86 miRNAs were obtained after eliminating duplicates (Figures 8A–C). A total of 1,632 TFs were screened out, the top 25 TFs were selected for visualization, and the TF–mRNA–miRNA network was created. We found that CADM3 was regulated by the hsa-miR-6721-5p and SCRT1 (Figure 8D; Supplementary Table S12). A total of 1,310 lncRNAs were forecasted; due to the excessive number of lncRNAs predicted in this study, the ceRNA network is shown in Supplementary Table S13. Therefore, the miRNA–mRNA network was created, and hsa-miR-5193 regulated CADM3 and GPR78; moreover, CADM3 and GPR78 were all regulated by hsa-miR-4290 (Figure 8E).

### 3.8 The drug prediction

**Figure 9:**
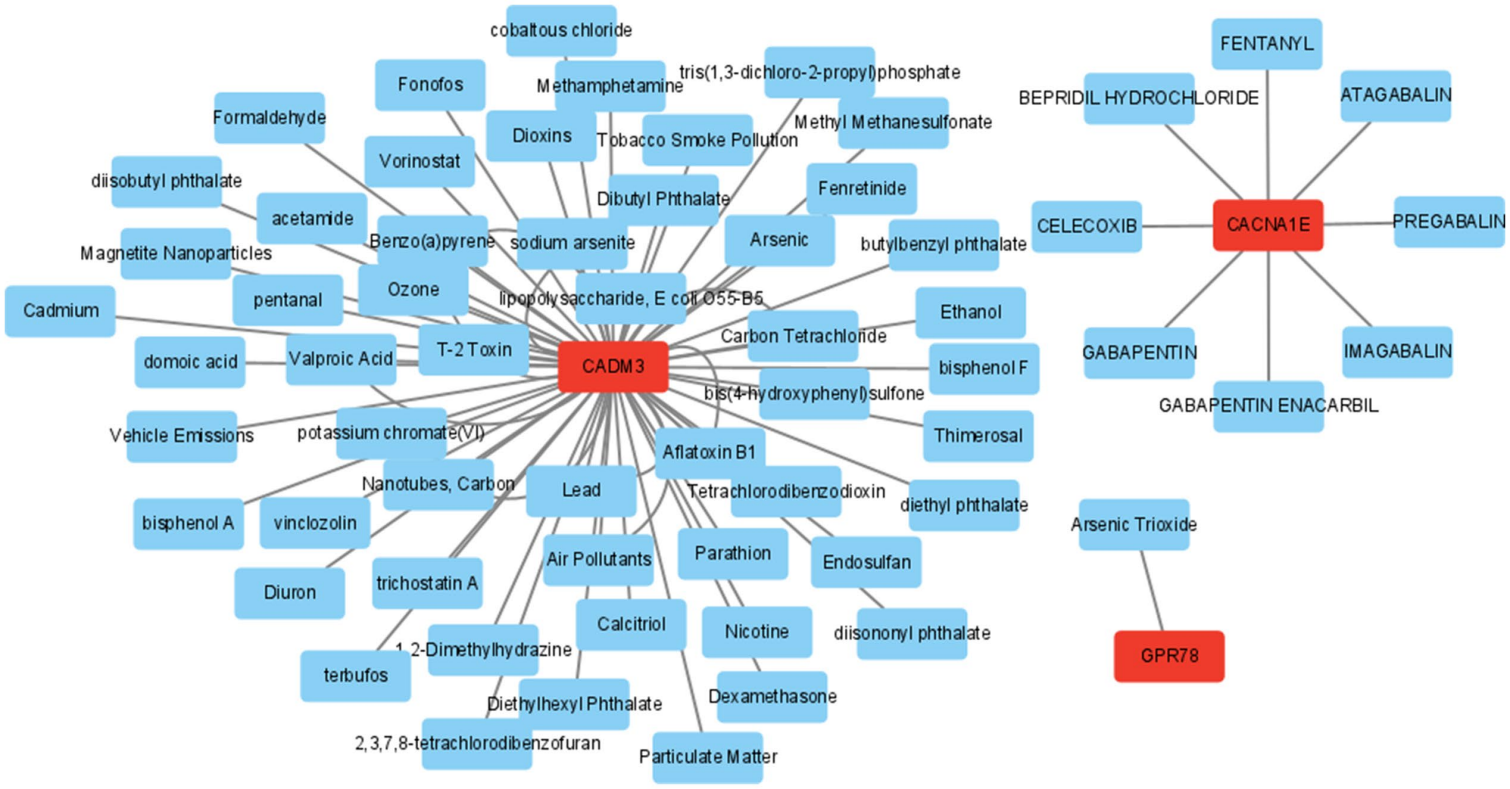
Drug prediction network diagram. Red is mRNA; blue is the predictive drug.

In addition, the mRNA–drug network (65 nodes and 71 edges) was created, including “PREGABALIN,” “1,2-Dimethylhydrazine,” and “Arsenic Trioxide” (Figure 9; Supplementary Table S14).

### 3.9 The verification of biomarkers by qRT-PCR

**Figure 10:**
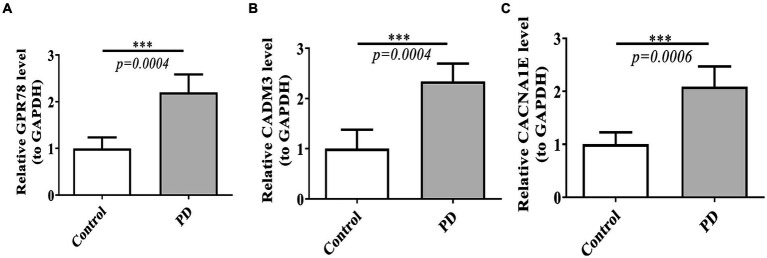
Verification of three DEGs by qT-PCR between PD and control groups. **(A)** Expression of GPR78 between PD and control groups. **(B)** Expression of CADM3 between PD and control groups. **(C)** Expression of CACNA1E between PD and control groups. GPR 78, orphan G protein-coupled receptor 78; CADM3, cell adhesion molecule 3; qRT-PCR, quantitative simultaneous polymerase chain reaction.

Power analysis manifested that the sample size required for a single group was 4 (Supplementary Table S15). Based on the qRT-PCR verification results, we found that GPR78, CADM3, and CACNA1E were up-regulated in the PD group, and the validation results were consistent with the above analyses (Figures 10A–C).

## 4 Discussion

Parkinson’s disease (PD) is a progressive neurodegenerative disorder pathologically characterized by the loss of dopaminergic neurons in the substantia nigra and the presence of protein inclusions termed Lewy bodies (Pajares et al., 2020). NETs are involved in numerous pathological processes, including infection (He et al., 2022), autoimmune diseases (Gupta et al., 2022), tumor development (Tamura et al., 2022), Alzheimer’s disease (Pietronigro et al., 2017), acute ischemic stroke (Vallés et al., 2017), peripheral nerve injury (Tansley et al., 2022), thrombosis (Zhou et al., 2022), and NMDA encephalitis (Qiao et al., 2022; Zhou et al., 2022). However, its role in PD remains unclear. Here, we conducted bioinformatics gene analysis for potential biomarkers and therapeutic targets of PD based on neutrophil extracellular traps. A total of three biomarkers including GPR78 (orphan G protein-coupled receptor 78), CADM3 (cell adhesion molecule 3), and CACNA1E were identified through bioinformatics gene analysis.

GPR78 is a member of the G protein-coupled receptor (GPCR) family, which represents the most abundant group of cell surface receptors. Several members of this family have already been implicated in the pathophysiology of PD. G protein-coupled receptors, including dopamine receptors, play a pivotal role in regulating multiple intracellular signaling pathways, thus modulating the functionality of neuronal circuits affected by PD. In addition to dopamine receptors, several other GPCRs are also capable of regulating the neural circuits affected by PD, and many are currently being investigated as potential therapeutic targets for various aspects of PD. For example, researchers found that the deletion of GPR6 in mice leads to a decrease in striatal cAMP levels, an increase in locomotor activity, and slight reductions in L-DOPA or dopamine agonist-induced dyskinesia (Oeckl et al., 2014). Except for GPR6, the study also found the levels of ecto-GPR37 in the cerebrospinal fluid of PD patients were significantly higher. Moreover, CSF ecto-GPR37 demonstrated superior diagnostic performance for PD than total α-synuclein (Morató et al., 2021). As for GPR78, it was identified by virtue of its homology to orphan GPR26, which was identified in humans and rats (Lee et al., 2000, 2001) The expression of GPR78 mRNA in the pituitary and placenta suggests its potential involvement in the functioning of the hypothalamic–pituitary–adrenal (HPA) axis and pregnancy (Lee et al., 2001). However, until now, no studies have investigated the relationship between GPR78 and PD. Studies have shown that the GPR78 gene is linked to bipolar affective disorder (BPAD) and schizophrenia in a large Scottish family (Underwood et al., 2006). In particular, the neurodegeneration observed in PD is not confined solely to dopaminergic neurons, and patients also experience non-motor symptoms such as cognitive impairment or neuropsychiatric disturbances. The potential involvement of GPR78 in psychiatric symptoms among PD patients warrants further investigation to enhance our understanding. The aforementioned statement serves as a reminder that GPR78 may also play a role in the pathogenesis of PD, necessitating further experimentation to elucidate this association in subsequent studies.

The CADM family of proteins also referred to as nectin-like (Necl) and synaptic cell adhesion (SynCAM) molecules. The CADM protein family comprises four neuron-specific adhesion molecules (CADM1, CADM2, CADM3, and CADM4), CADM1 (Necl2), CADM2 (Necl3), and CADM3 (Necl1) are found in axons, while CADM2 and CADM4 (Necl4) are present in myelinating Schwann cells (Maurel et al., 2007; Spiegel et al., 2007). The malfunctioning of normal CAM is suspected to contribute to synaptic dysfunction, potentially leading to neurodegeneration. As for AD, Necl-1 expression is significantly upregulated in pyroglutamate-modified amyloid β-expressing transgenic mice (TBA42 mice) (Yang et al., 2013). These results suggest that nectins and Necl-1 are implicated in the pathology of AD. A genome-wide association scan in Sardinians revealed that an inflammatory biomarker, monocyte chemotactic protein-1 (MCP-1), was associated with the SNP in CADM3 (rs3845624) (Naitza et al., 2012). In a model of depressive behavior after the immune challenge, cell adhesion molecule 3 (Cadm3) on microglia was over-expressed (Gonzalez-Pena et al., 2016). Currently, there is a dearth of research on the association between CADM3 and PD, and the underlying mechanism by which CADM3 contributes to PD remains elusive. The onset of PD is widely acknowledged to be closely associated with microglia-mediated inflammation. Previous studies have also indicated the involvement of CADM3 in the adhesion of inflammatory cells to endothelial cells and microglia-mediated inflammation. Our study further suggests that CADM3 may contribute to the development of PD through neutrophil extracellular traps (NETs). However, more comprehensive research is required to validate this hypothesis.

In our study, bioinformatics gene analysis found another key gene biomarker of NETs for PD was CACNA1E. The CACNA1E gene exhibits robust expression in the central nervous system and encodes the alpha-1 subunit of the voltage-gated CaV2.3 channel, which mediates high voltage-activated R-type calcium currents that initiate synaptic transmission (Williams et al., 1994; Wormuth et al., 2016). The influx of Ca2+ plays a crucial role in modulating neuronal excitability and activating Ca2 + −dependent physiological processes. However, the rhythmic Ca2+ load in SN DA neurons also induces mitochondrial oxidative stress. Disrupted Ca2+ homeostasis and mitochondrial dysfunction are regarded as pivotal factors in the pathophysiology of PD (Guzman et al., 2010; Zaichick et al., 2017; Zampese and Surmeier, 2020). Researchers found that the Cav2.3 subtype of voltage-gated Ca2+ channels exhibits the highest level of expression in adult SN dopaminergic neurons and levels increase with age (Benkert et al., 2019). In MPTP-induced PD mouse model, global Cav2.3 knockout fully prevented SN DA neuron degeneration and profoundly reduced somatic Ca2+ oscillations (Benkert et al., 2019). In a microbiota-induced depression animal model, proteomic analysis of the olfactory bulb suggests CACNA1E and its downstream CREB signaling were down-regulated, which provides a novel insight for further research of the “microbiota-gut-brain axis” (Huang et al., 2019). PD patients also have a decreased sense of smell, and its pathogenesis is related to the abnormal function of the microbial–gut–brain axis, and whether CACNA1E is involved in this process needs to be further studied.

According to the GO enrichment analysis, we found that GPR78 and CACNA1E mainly participated in the ‘hydrogen peroxide catabolic process’ and ‘haptoglobin hemoglobin complex’. KEGG enrichment analysis demonstrated that GPR78, CADM3, and CACNA1E were mainly associated with ‘cell cycle’ and ‘glycine serine and threonine metabolism’. Studies conclude that the degeneration and loss of nerve cells in PD may be attributed to the direct generation of hydrogen peroxide during extracellular or intracellular protein aggregation, leading to oxidative damage, particularly in the presence of metals (Tabner et al., 2002). Hydrogen peroxide is a member of reactive oxygen species (ROS), the enzyme superoxide dismutase catalyzes the conversion of superoxide into hydrogen peroxide, which is subsequently degraded by either catalase or glutathione peroxidase (von Ossowski et al., 1993). Experiments concluded that the α-synuclein molecule inhibited the expression of catalase; thus, the low catalase activity and high hydrogen peroxide production lead to PD (Graham, 1978; Yakunin et al., 2014). Based on the previous literature reports and our findings, it is postulated that the three co-DEGs we have identified may exert an influence on the pathogenesis of PD via the oxidative stress mechanism mediated by hydrogen peroxide.

Bioinformatics gene analysis on DEGs in the Gene Expression Omnibus (GEO) database (GSE8397 and GSE22491) of PD patients found that Ankyrin 1 (ANK1) was the only common gene differentially down-regulated in lateral substantia nigra (LSN), medial substantia nigra (MSN), and blood. GO analysis displayed that these DEGs were mainly enriched in hemoglobin complex, haptoglobin–hemoglobin complex and cortical cytoskeleton, and so on (Xue et al., 2023), which was consistent with our results. The modulation of iron homeostasis may be influenced by hemoglobin, which serves as the primary source of peripheral iron. Furthermore, an association has been suggested between increasing levels of hemoglobin and a significant rise in the incidence of PD (Abbott et al., 2012). The dysregulation of iron homeostasis in patients with PD has been demonstrated by several studies (Savica et al., 2009). The increased iron level has been found in the SN in PD patients in comparison with controls and high iron concentrations may be responsible for PD pathogenesis (Sengstock et al., 1993). Our results are consistent with the findings presented herein, providing evidence that GPR 78 and CACNA1E have the potential to serve as a valuable biomarker for facilitating the diagnosis of Parkinson’s disease.

In our study, KEGG enrichment analysis demonstrated the co-DEGs were associated with ‘glycine, serine, and threonine metabolism’. Several studies have consistently revealed that the pathophysiology of PD is deeply associated with amino acid metabolism, which has emerged as a distinctive biological hallmark characterizing PD. Glycine serves as a co-agonist with glutamate at the site of glutamate receptors, functioning as an additional inhibitory neurotransmitter. Inhibiting glycine transport can enhance the activity of dopamine axons (Schmitz et al., 2013), which supported our results. The study also reported there was a significant difference in threonine concentrations between the early-stage PD patients and advanced-stage PD patients with levodopa-induced dyskinesia and threonine concentrations correlated with disease duration, but not with levodopa equivalent dose taken daily (Figura et al., 2018). A recent study conducted by LeWitt et al. reported that alterations in serine and taurine concentrations exhibited the strongest predictive capability for changes in the UPDRS II + III score through analysis of the baseline 15 plasma compounds of PD patients (LeWitt et al., 2017). The literature is consistent with our findings, suggesting that the key genes (GPR78, CADM3, and CACNA1E) identified in our study are promising markers for predicting PD.

Immune infiltration analysis between PD and control groups in our study showed that five different kinds of differential immune cells, respectively, were resting mast cells, macrophages M0, monocytes, naive B cells, and activated NK cells. Microglia are CNS-resident macrophages, initially described by Pio del Rio Ortega (Del Río-Hortega Bereciartu, 2020). The injection of 6-OHDA in rats induces a reactive microgliosis that precedes the initiation of astrogliosis and dopaminergic cell death. The chronically activated microglia secrete elevated levels of proinflammatory mediators, which inflict damage upon neurons and further stimulate microglia, thereby establishing a self-perpetuating cycle that promotes inflammation and neurodegeneration. Studies demonstrated that peripheral inflammation was involved in PD origin and spreading. For example, oxidative modification of α-Syn generates novel antigenic epitopes capable of initiating peripherally driven CD4+ and CD8+ T-cell responses (Benner et al., 2008). On the other hand, in the murine model of intra-striatal injection of preformed fibril (PFF) α-Syn, except for microglial and astrocyte activation, there is significant infiltration of B, CD4+ T, CD8+ T, and natural killer cells (Earls et al., 2019). Our study provides some evidence for the involvement of NETs in PD, and further research is imperative to unravel the intricate involvement of the peripheral immune system in disease initiation and propagation toward the central nervous system.

In the current study, hsa-miR-4290 and hsa-miR-5193 were predicted and confirmed as a downstream target of CADM3 and GPR 78; hsa-miR-6721-5p and SCRT1 were also predicted as a downstream target of CADM3. SCRT1 is a recently identified transcriptional repressor belonging to the SNAIL family of zinc finger transcription factors. Xia et al. found that epigenome-wide DNA methylation analysis of whole blood cells showed differential expression of CRT1 among individuals with generalized anxiety disorder (GAD), obsessive-compulsive disorder (OCD), and healthy controls. This suggests that CRT1 may help distinguishing patients from healthy controls or classifying patients with GAD and OCD (Guo et al., 2022). SCRT1 also regulated the conversion from microglia to neurons, which was crucial for nerve system development (Malygina et al., 2021). It is well known that patients with PD often have emotional problems and that PD is associated with microglia and neuroinflammation. Although there was no research on the PD population, our study and previous literature may provide a basis for further exploration of the mechanism of SCRT1 in PD patients. To date, there have been no studies on hsa-miR-4290 and hsa-miR-5193 in PD. However, according to bioinformatics gene data from TargetScan, miR-5193 was predicted to target TRIM11 (Pan et al., 2019) and early published studies have identified TRIM11 involvement in neurodegenerative disorders (Lee et al., 2013) as well as in the regulation of Alzheimer’s disease by destabilizing intracellular humanin (Niikura et al., 2003). Our study also predicted hsa-miR-5193 involved in the onset of PD, which needs more research to validate this conclusion. A study of COVID-19 found that miR-6721-5p was involved in inflammatory pathways, the latter was also one of the main mechanisms of PD, and our study on NETs in PD patients also found the same result (Hashemi Sheikhshabani et al., 2023).

A deeper understanding and accurate detection of the immunological mechanisms underlying the earliest signs of Parkinsonism will lead to new therapies and in future may enable clinicians to intervene effectively with novel or repurposed anti-inflammatory and immunomodulatory therapies to slow or delay the progression of disease from the periphery to the CNS. Our study aimed to provide biomarkers that can be used for early diagnosis and disease-modifying therapy of PD by exploring extranuclear neutrophil trapping net genes associated with inflammation in a PD patient database.

## 5 Strength and limitations

Our study is the first to identify common differentially expressed genes in PD and NETs through bioinformatics gene analysis. Additionally, we explore the enrichment pathways of these co-DEGs, inflammatory cell infiltration, and downstream RNA pathways and predict potential intervention drugs. Furthermore, we validated the nomogram model established by co-DEGs in other databases as well as in blood samples from both PD patients and control populations to enhance the reliability of our experimental results. The drawback of our study lies in the fact that it is based solely on microarray analysis, which relies on gene expression values. However, as gene expression may not directly correlate with protein expression, the biomarkers identified in this study should be considered at the gene level, rather than at the protein level. Validation should be conducted through both *in vitro* and *in vivo* experiments and clinical trials. Moreover, to some extent, larger prospective clinical studies would provide a more comprehensive validation of our findings.

## 6 Conclusion

Our study reported co-DEGs of GPR78, CADM3, and CACNA1E link NETs and Parkinson’s disease and established a nomogram model to diagnose PD based on these genes, which also performed well in external cohort validation. GO and KEGG enrichment analysis of the key genes showed the co-DEGs mainly participated in the ‘hydrogen peroxide catabolic process’, ‘haptoglobin hemoglobin complex’, and ‘glycine serine and threonine metabolism’. By further immune infiltration analysis between PD and control groups, we found that co-DEGs were associated with five kinds of immune cells. Finally, the top miRNAs for each co-DEG may be potential biomarkers or therapeutic targets for NETs-PD, especially hsa-miR-5193 and hsa-miR-4290. In addition, we examined co-DEGs in blood samples of both PD and control patients, which showed that the co-DEGs in PD patients were higher than those in the control population. Thus, there is an association between NETs and PD, and expression of GPR78, CADM3, and CACNA1E genes could serve as a biomarker for NET-related PD.

## Data availability statement

The raw data supporting the conclusions of this article was downloaded from the public database which is detailed in the methods section of this article. Data for subsequent qT-PCR validation can be obtained by contacting the corresponding author.

## Ethics statement

The studies involving humans were approved by the First Hospital of Lanzhou University ethics committee. The studies were conducted in accordance with the local legislation and institutional requirements. The participants provided their written informed consent to participate in this study.

## Author contributions

XW: Conceptualization, Data curation, Formal analysis, Funding acquisition, Investigation, Methodology, Validation, Writing – review & editing. QW: Data curation, Methodology, Resources, Software, Writing – review & editing. YG: Formal analysis, Funding acquisition, Project administration, Validation, Writing – review & editing. JC: Supervision, Visualization, Writing – review & editing. XL: Data curation, Formal analysis, Software, Writing – original draft. CX: Data curation, Software, Writing – review & editing.

## Footnotes

^1^http://mirdb.org

^2^https://mirmap.ezlab.org/

^3^http://mirwalk.uni-hd.de/

^4^https://amp.pharm.mssm.edu/ChEA3

^5^http://starbase.sysu.edu.cn/

^6^http://ctdbase.org/

## Glossary

AD: Alzheimer’s disease
BBB: Blood–brain barrier
BPAD: Bipolar affective disorder
CADM3: Cell-adhesion molecule 3
CREB: cAMP-response element-binding protein
CSF: Cerebrospinal fluid
DEG: Differentially expressed gene
DMNC: Density of maximum neighborhood component
EPC: Edge percolated component
GO: Gene ontology
GPCR: G protein-coupled receptor
GPR 78: Orphan G protein-coupled receptor 78
HPA: Hypothalamic pituitary adrenal
KEGG: Kyoto encyclopedia of genes and genomes
LB: Lewy bodies
LSN: Lateral substantia nigra
MCC: Maximal clique centrality
MCP-1: Monocyte chemotactic protein-1
MPTP: 1-methyl-4-phenyl-1,2,3,6-tetrahydropyridine
MNC: Maximum neighborhood component
NET: Neutrophil extracellular traps
NK: Natural killer cells
PBMC: Peripheral blood mononuclear cells
PD: Parkinson’s disease
qRT-PCR: Quantitative simultaneous polymerase chain reaction
RA: Rheumatoid arthritis
ROS: Reactive oxygen species
SCRT1: Scratch family transcriptional repressor 1
SLE: Systemic lupus erythematosus
TF: Transcription factor
