# Supplementary material for: Bioinformatics gene analysis for potential biomarkers and therapeutic targets of Parkinson’s disease based on neutrophil extracellular traps
Source: Front Aging Neurosci. 2024 May 31;16:1388226. doi: 10.3389/fnagi.2024.1388226 (PMC11178047; doi:10.3389/fnagi.2024.1388226)
Supplement: Supplementary file 15 [file Table_15.DOCX]

Supplementary Table 15 Power analysis

| **y** | **Group 1** | **Group 2** | **effsize** | **n** | **Sig.level** | **Power** |
| --- | --- | --- | --- | --- | --- | --- |
| CADM3 | Control PBMC samples | PD PBMC samples | -5.90 | 2.13 | 0.05 | 0.9 |
| CACNA1E | Control PBMC samples | PD PBMC samples | -4.80 | 2.39 | 0.05 | 0.9 |
| GPR78 | Control PBMC samples | PD PBMC samples | -3.23 | 3.34 | 0.05 | 0.9 |
